# Testing *Fmr1*^*KO*^ Phenotypes in Response to GSK3 Inhibitors: SB216763 versus AFC03127

**DOI:** 10.3389/fnmol.2021.751307

**Published:** 2021-10-07

**Authors:** Pamela R. Westmark, Beatrice Garrone, Rosella Ombrato, Claudio Milanese, Francesco Paolo Di Giorgio, Cara J. Westmark

**Affiliations:** ^1^Department of Neurology, University of Wisconsin, Madison, WI, United States; ^2^Angelini Pharma S.p.A., Rome, Italy; ^3^Molecular and Environmental Toxicology Center, University of Wisconsin, Madison, WI, United States

**Keywords:** fragile X syndrome, glycogen synthase kinase, amyloid precursor protein, metabotropic glutamate receptor 5, seizure

## Abstract

Glycogen synthase kinase 3 (GSK3) is a proline-directed serine-threonine kinase that is associated with several neurological disorders, including Alzheimer’s disease and fragile X syndrome (FXS). We tested the efficacy of a novel GSK3 inhibitor AFC03127, which was developed by Angelini Pharma, in comparison to the metabotropic glutamate receptor 5 inhibitor 2-Methyl-6-(phenylethynyl)pyridine hydrochloride (MPEP) and the GSK3 inhibitor SB216763 in *in vivo* and *in vitro* assays in *Fmr1*^*KO*^ mice, a mouse model useful for the study of FXS. The *in vivo* assay tested susceptibility to audiogenic-induced seizures (AGS) whereas the *in vitro* assays assessed biomarker expression and dendritic spine length and density in cultured primary neurons as a function of drug dose. MPEP and SB216763 attenuated AGS in *Fmr1*^*KO*^ mice, whereas AFC03127 did not. MPEP and AFC03127 significantly reduced dendritic expression of amyloid-beta protein precursor (APP). All drugs rescued spine length and the ratio of mature dendritic spines. Spine density was not statistically different between vehicle and GSK3 inhibitor-treated cells. The drugs were tested over a wide concentration range in the *in vitro* assays to determine dose responses. A bell-shaped dose response decrease in APP expression was observed in response to AFC03127, which was more effective than SB216763. These findings confirm previous studies demonstrating differential effects of various GSK3 inhibitors on AGS propensity in *Fmr1*^*KO*^ mice and confirm APP as a downstream biomarker that is responsive to GSK3 activity.

## Introduction

**FIGURE 1:**
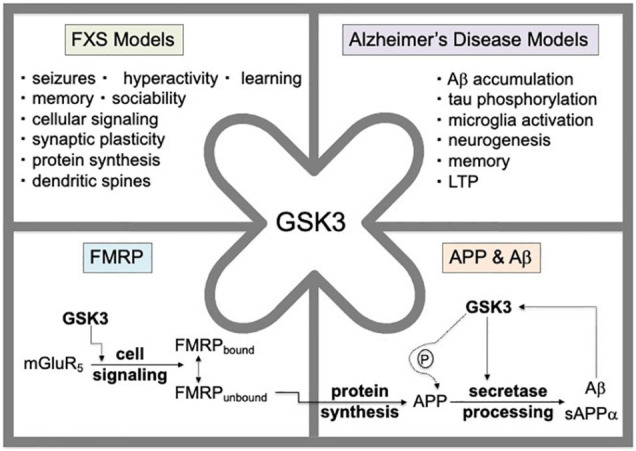
GSK3 activation affects all major hallmarks of Alzheimer’s disease and FXS. GSK3 signals downstream of mGluR_5_ and upstream of FMRP. The mGluR_5_/FMRP signaling pathway modulates the cellular protein synthesis of numerous FMRP target mRNAs including *App* mRNA. Genetic reduction of *App* by 50% in *Fmr1*^*KO*^ mice rescues behavioral, dendritic spine and electrophysiological phenotypes. GSK3 modulates APP and Aβ levels through phosphorylation of the carboxy-terminus of APP and via regulation of secretase enzymes involved in amyloidogenic processing of APP. The Aβ metabolite of APP can induce GSK3 activity suggesting a positive feedback regulatory loop.

Fragile X syndrome (FXS) is a trinucleotide repeat disorder associated with the loss of expression of fragile X mental retardation protein (FMRP) and subsequent intellectual disability, autism, hyperactivity and seizures (Hagerman and Hagerman, 2002). FMRP is an RNA binding protein that regulates the protein synthesis of numerous synaptic proteins, including amyloid-beta precursor protein (APP; Westmark and Malter, 2007; Lee et al., 2010). Altered levels of APP and its metabolites are found in *Fmr1*^*KO*^ mice, a mouse model that lacks expression of FMRP, as well as in subjects with autism and FXS (Westmark and Malter, 2007; Ray et al., 2011, 2016; Westmark et al., 2011). Genetic reduction of *App* in *Fmr1*^*KO*^ mice rescues behavioral, dendritic spine and electrophysiological phenotypes (Westmark et al., 2011, 2016). Furthermore, drugs that inhibit metabotropic glutamate receptor 5 (mGluR_5_), which signals upstream of FMRP, reduce dendritic overexpression of APP in *Fmr1*^*KO*^ primary cultured neurons (Westmark et al., 2018) and seizures in mouse models that over-express human APP and amyloid-beta (Aβ; Westmark et al., 2009, 2010). Thus, APP is both a potential therapeutic target as well as a disease biomarker for FXS (Westmark, 2019). Glycogen synthase kinase 3 (GSK3) signals downstream of mGluR_5_ and upstream of FMRP, and its activation affects all major hallmarks of Alzheimer’s disease and FXS (Figure 1). Herein, we sought to determine the efficacy of a novel GSK3 inhibitor developed by Angelini Pharma in attenuating *in vivo* (audiogenic-induced seizures, AGS) and *in vitro* (dendritic APP expression, spine length, ratio of mature dendritic spines to filopodia) phenotypes in *Fmr1*^*KO*^ mice.

Glycogen synthase kinase 3 is a constitutively active serine-threonine protein kinase that signals through numerous cellular signaling pathways including mGluR_5_ (Portis et al., 2012). Phosphorylation of protein substrates by GSK-3 usually results in inhibition of those proteins. GSK3 has a bi-lobar structure including the N-terminus that binds to ATP and the C-terminus, or activation loop, that mediates the kinase activity. GSK3 activity is elevated in *Fmr1*^*KO*^ mouse brain where inhibition of mGluR_5_ leads to inhibition of GSK3 indicating coordinated regulation of two key signaling proteins (Min et al., 2009). In 1996, Klein and Melton (1996) determined that the GSK3 inhibitor lithium rescues long-term memory in *dfmr1* flies. Subsequent studies with lithium confirmed these findings in flies as well as demonstrated rescue of AGS, hyperactivity, social deficits, anxiety, learning and memory, cerebral protein synthesis, dendritic spine phenotypes, neurogenesis, and mGluR-LTD in *Fmr1*^*KO*^ mice (McBride et al., 2005; Min et al., 2009; Choi et al., 2010, 2011, 2016; Mines et al., 2010; Yuskaitis et al., 2010; Liu et al., 2011, 2012; Guo et al., 2012; Chen et al., 2013; King and Jope, 2013). Pharmaceutical GSK3 inhibitors also rescue a wide range of pathophysiological features in *Fmr1*^*KO*^ mice (Min et al., 2009; Luo et al., 2010; Mines et al., 2010; Guo et al., 2012; King and Jope, 2013; Chen et al., 2014; Franklin et al., 2014; King et al., 2014; Pardo et al., 2017; McCamphill et al., 2020). GSK3 inhibitors are under study for the treatment of numerous disorders, including Alzheimer’s disease, autism spectrum disorders and type 2 diabetes mellitus; and the preclinical data, in conjunction with a pilot clinical trial of lithium in FXS, suggest that GSK3 inhibitors are a potential treatment strategy for FXS (Berry-Kravis et al., 2008; Mines and Jope, 2011; Hagerman et al., 2012; Liu and Smith, 2014; Rizk et al., 2021).

Preclinical validation of novel GSK3 inhibitors is necessary to progress the most effective compounds into clinical trials (Roca and Campillo, 2020). Angelini Pharma identified novel inhibitors of GSK3 through a structure-based hit discovery process (Furlotti et al., 2015; Ombrato et al., 2015). The compounds were synthesized as described in the patent application WO2013124158A1 and evaluated for pharmacokinetic properties after intraperitoneal administration in C57BL/6J mice (Furlotti et al., 2015). Based on these data, this study compared the efficacy of the initial lead compound AFC03127 with the mGluR_5_ inhibitor 2-Methyl-6-(phenylethynyl)pyridine hydrochloride (MPEP) and the GSK3 inhibitor SB216763 in *Fmr1*^*KO*^ mice with the goal of identifying a more efficacious compound indicated for FXS. SB216763 is a commercially available maleimide derivative with potent and selective cell permeable, ATP competitive inhibitor activity of GSK3 (*K*i = 9 nM; Coghlan et al., 2000). AFC03127 is a 5-substituted-N-(piperidin-4-ylmethyl)-1H-indazole-3-carboxamide compound with reversible, linear, ATP-competitive GSK3 inhibitor activity (*K*i = 15 nM; Furlotti et al., 2015). AFC03127 exhibits an IC_50_ of 40 nM for GSK3α and an IC_50_ of 18 nM for GSK3β in *in vitro* ATPase enzymatic assays as well as an AUC_*o,inf*_ of 370 ± 96 μg.min/mL, *C*_*max*_ of 2.1 ± 0.7 μg/mL, *T*_*max*_ of 15 min, *t_1__/__2_* of 203 ± 53 min, *Vd* of 6 ± 2 L/kg, and CI of 0.02 ± 0.01 L/min/kg by *in vivo* pharmacokinetic testing of plasma from mice dosed at 10 mg/kg I.P. with a *C*_*max*_ of 5.5 ± 0.4 ng/mg protein in brain (Furlotti et al., 2015). Additional preclinical GSK3 ATPase enzymatic assays indicate IC_50_ values of 7.54 nM (SB216763, GSK3α), 21.3 nM (SB216763, GSK3β), 12 nM (AFC03127, GSK3α), and 12.3 nM (AFC032127, GSK3β; Angelini, unpublished data). AFC03127 is under development as a mood stabilizing drug and is expected to have superior activity to commercially available SB216763 based on high plasma stability and its potent ATP-competitive inhibitor activity (Furlotti et al., 2015; Capurro et al., 2020). AFC03127 is identified as 14i and AF3581, respectively, in the cited references. We hypothesize that AFC03127 will reduce AGS in *Fmr1*^*KO*^ mice as well as dendritic APP expression, spine length and the percent of filopodia in *Fmr1*^*KO*^ cells.

## Materials and Methods

### Test Compounds

MPEP was purchased from Tocris (catalog #1212, Minneapolis, MN, United States). SB216763 was purchased from SpiroChem (Basel, Switzerland). AFC03127 was provided by Angelini Pharma S.p.A. (Rome, Italy). For the *in vivo* work, the test compounds were prepared as a fine suspension in 0.5% hydroxypropyl methylcellulose (HPMC) using an IKA-Ultra Turrax mill. For the *in vitro* work, the test compounds were dissolved in a small volume of dimethyl sulfoxide (DMSO), serially diluted in DMSO, and then diluted in Hank’s buffered salt solution (HBSS) prior to treating the neuronal cells, such that the final concentration of DMSO in the cell media was 0.5%. The higher GSK3 inhibitor drug concentrations (25 and 250 μM) formed suspensions when the DMSO stocks were diluted with HBSS, with SB216763 visibly more insoluble than the AFC03127. Persons conducting the experiments were blinded to the identity of the compounds until after data acquisition and analysis.

### Animal Husbandry

The *Fmr1*^*KO*^ mice were originally developed by the Dutch-Belgian FXS Consortium (Dutch-Belgian Fragile X Consortium, 1994) and backcrossed into and maintained in the C57BL/6J background (Dr. Bill Greenough’s laboratory, University of Illinois at Urbana-Champaign). The *Fmr1*^*KO*^ mice have been maintained at the University of Wisconsin-Madison for over 17 years with occasional backcrossing with C57BL/6J mice from Jackson Laboratories to avoid genetic drift. Mice were socially housed in static microisolator cage on a 6 a.m.–6 p.m. light cycle with *ad libitum* access to food (Teklad 2019 mouse diet) and water. The breeding scheme for this study included crossing *Fmr1*^*KO*^ female with *Fmr1*^*KO*^ male mice, and offspring of both sexes were used for experiments. All animal husbandry and euthanasia procedures were performed in accordance with an approved University of Wisconsin-Madison animal care protocol administered through the Research Animal Resources Center with oversight from the Institutional Animal Care and Use Committee. *Fmr1* genotypes were determined by PCR analysis of DNA extracted from tail biopsies.

### Audiogenic-Induced Seizures

Individual mice were randomized to drug treatment groups (all treatment cohorts were derived from a minimum of 4 litters of mice). *Fmr1*^*KO*^ mice in the C57BL/6J background have peak sensitivity to AGS at postnatal day 21 (P21; Yan et al., 2004). Thus, mice were treated with vehicle or the indicated dose of drug by intraperitoneal (I.P.) injection at P21 and 30 min later transferred to a Plexiglas box (13″L X 8″W X 7″H) and exposed to a high-pitched siren (118 dB) from a personal body alarm (LOUD KEY^TM^) for 3 min. The number of mice exhibiting wild running (WR), tonic-clonic seizures (AGS) and death were scored. The treatment groups included: (1) 0.5% HPMC vehicle, (2) 30 mg/kg MPEP, (3) 10 mg/kg AFC03127, and (4) 30 mg/kg SB216763. Males and females were randomized to treatment groups. A dosing volume of 20 mL/kg was used for I.P. and took into consideration the established concentration of MPEP known to reduce AGS. GSK inhibitor doses were selected based on prior Angelini pharmacokinetic and behavior data (Capurro et al., 2020; Angelini, personal communication). A lower dose of AFC03127 (10 mg/kg) was used compared to SB216763 (30 mg/kg) because prior behavior data indicated a sedative effect with 30 mg/kg AFC03127. Average mouse weight was 8.7 ± 1.4 (SD) grams.

### Preparation and Treatment of Primary Cultured Neurons

Pregnant female *Fmr1*^*KO*^ mice (embryonic day 18) were anesthetized with isoflurane prior to decapitation and transfer of the uterine sac to ice-cold HBSS. Cortices were removed, washed with ice-cold HBSS, lysed with 0.5 mg/mL trypsin for 25 min at 37°C, washed with HBSS (Gibco Life Technologies, catalog #14170161), suspended in NeuroBasal medium (Gibco Life Technologies, catalog #21103-049; supplemented with 2% B27 supplement, penicillin/streptomycin, 0.5 mM glutamine), triturated 70x with a 10 mL pipet and passed through a 70 μm cell strainer. Cells were counted by trypan blue dye exclusion, plated at 1 × 10^5^ cells/mL on poly(D)-lysine coated glass coverslips in 12-well tissue culture dishes, and cultured for 18 days (APP staining) or 16 days (DiI staining) at 37°C/5% CO_2_. Cells were treated with the indicated doses of drug in NeuroBasal culture media containing B27 supplement for the indicated times. Tested concentrations of drugs spanned a range of 2.5 nM–250 μM for the GSK3 inhibitors. MPEP was tested at 25 μM, an established concentration known to reduce dendritic APP levels and spine length. The cells were dosed *in vitro* at a constant dose volume of 1 mL solution per well.

### APP Staining, Confocal Microscopy and Image Analysis

To assess dendritic APP levels, treated neuronal cells were fixed and stained with anti-APP antibody (anti-22C11, catalog number mAB348-AF647, EMD Millipore Corporation, Temecula, CA, United States). Anti-22C11 is a well-validated monoclonal antibody that targets amino acids 66–81 in the amino terminus of APP. For fixation, treated cells were washed with Dulbecco’s phosphate buffered saline (DPBS, Gibco Life Technologies, catalog #14190250), fixed in 4% paraformaldehyde for 10 min at room temperature and permeabilized with methanol (–20°C) for 15 min. Fixed, permeabilized cells were stained with anti-22C11 conjugated with Alexa Fluor^®^ 647 (1:500, overnight). Controls with no primary antibody were run in parallel. Washes and antibody dilutions were in DPBS containing 2% fetal bovine serum. Coverslips were fixed to slides with 12 μL ProLong Gold Antifade (Invitrogen, Carlsbad, CA, United States) and dried overnight. Images were acquired with a Nikon A1RS HD Confocal Microscope System (with Eclipse Ti2-E/A inverted microscope) with high sensitivity gallium arsenide phosphide (GaAsP) detectors, resonant scanner, fluorescence spectral detection using the 405 and 638 nm lasers, Nikon Plan Apo 60x/1.40 oil objective with Fisher Scientific Immersion Oil at ambient temperature, Capture Z-series (Step: 0.3 μm, 13 steps, and Range: 3.3 μm) with the stacks summed, and Nikon NIS-Elements [v.4.50] and NIS-Elements Viewer [Version 4.2] software (Nikon Corp, Tokyo, Japan). Image resolution was 8.0453 pixels/micron (1,024 × 1,024). An average of 3-6 image stacks were acquired per coverslip and 3 coverslips were analyzed per treatment. APP levels in the puncta of 3–5 dendrites per image were quantitated with ImageJ (v. 1.53c) software using the Analyze Particles function (Rasband, W.S., Image J, U.S. National Institutes of Health, Bethesda, Maryland, United States, http://rsb.info.nih.gov/ij/, 1997–2006). A minimum of 2,238 puncta were analyzed per treatment. The average integrated density was calculated for each treatment and converted to a percentage compared to vehicle.

### Assessment of Dendritic Spine Length and Density

To assess dendritic spine phenotypes, treated neuronal cells were fixed with 4% paraformaldehyde and stained with DiI dye (Gibco Life Technologies, catalog #D282) as previously described (Westmark and Malter, 2007; Westmark et al., 2018). DiI is a lipophilic, orange-red fluorescent, membrane stain that diffuses laterally to stain the entire cell. Briefly, the wells were aspirated and sprinkled with DiI crystals and a small amount of DBPS was added to the edge of the wells to prevent dehydration of the cells. Cells were stained for 10 min, copiously washed with DPBS to remove all crystals and fixed to slides with ProLong Gold Antifade (Life Technologies Corporation, Carlsbad, CA, United States). Slides were allowed to set for at least 3 days to allow complete migration of the DiI into dendritic spines. Dendritic spines were imaged on a Nikon A1RS HD Confocal Microscope System as described above (excitation 562 nm DiI/excitation 403 nm DAPI) fitted with NIS-Elements software (v. 4.50) and using the Plan APO 100x/1.4 oil objective and Capture *Z*-series (Step: 0.3 μm, 13 steps, Range: 3.3 μm). The stacks were summed. Two coverslips were analyzed per treatment and 10 images of neurons were taken from multiple areas of each coverslip. All dendritic segments per image were quantitated. Spine length was quantitated with ImageJ using the R0I Manager Analysis function. Contours were drawn around the protrusions and the feret max (length) and feret min (widest width) of the contours were calculated. Spines (1,456–1,962) were quantitated per condition. The feret width was divided by feret max and protrusions having a ratio less than 0.5 were classified as filopodia and those with a ratio greater than or equal to 0.5 were classified as spines.

### Statistical Methods

Audiogenic-induced seizures treatment groups were compared by Fisher’s exact test for the analysis of contingency tables. Mice were randomized to treatment groups in roughly equal numbers of males and females, and there were not sex-specific differences by Fisher exact test so sexes were combined for the displayed analysis. Treatment groups for confocal microscopy of APP levels were compared by ANOVA and *post hoc* Dunnett’s multiple comparison tests for comparing all treatment groups to a control using Prism 9 software. Treatment groups for dendritic spine analyses were compared by ANOVA and *post hoc* Tukey tests for comparing the mean of all treatments to the mean of every other treatment to determine statistical significance for spine length and density using Prism 9 software and by Chi square analysis of contingency tables for percent filopodia. Statistical significance was defined as *p* < 0.05. The microscopist was blinded to treatment conditions.

## Results

**FIGURE 2:**
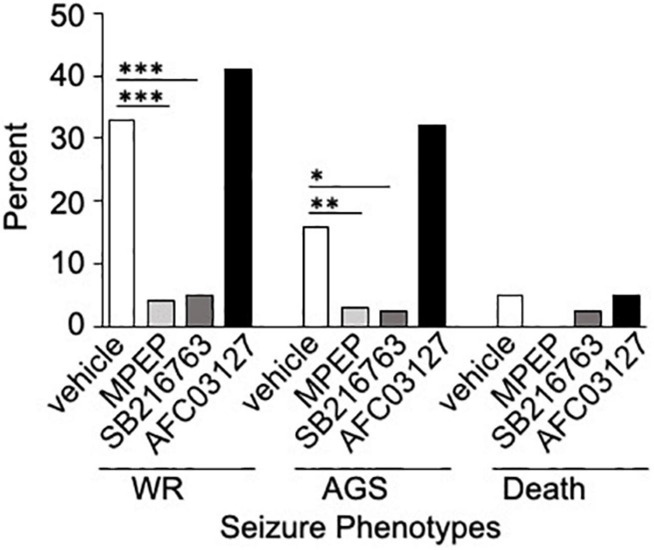
SB216763 but not AFC03127 inhibits AGS in *Fmr1*^*KO*^ mice. *Fmr1*^*KO*^ mice were treated with 0.5% HPMC vehicle, 30 mg/kg MPEP, 30 mg/kg SB216763, or 10 mg/kg AFC03127 I.P. at P21 and 30 min later tested for susceptibility to wild running (WR), tonic-clonic seizures (AGS), and death in the AGS assay. Treatment groups were compared to vehicle by Fisher’s exact test. There were a minimum of *n* = 22 mice per cohort [*n* = 159 vehicle (72 female and 87 male), *n* = 69 mice MPEP (32 female and 37 male), *n* = 40 mice SB216763 (11 female and 29 male), and *n* = 22 mice AFC03127 (10 female and 12 male)]. Asterisks indicate statistical significance of *p* ≤ 0.0001 (^∗∗∗^), *p* ≤ 0.01 (^∗∗^), and *p* ≤ 0.05 (^∗^).

Inhibitors of GSK3 are under investigation for the treatment of numerous disorders including Alzheimer’s disease, type 2 diabetes mellitus and FXS. Herein, we compared the efficacy of a novel Angelini Pharma GSK3 inhibitor (AFC03127) with established inhibitors of mGluR_5_ (MPEP) and GSK3 (SB216763; Coghlan et al., 2000; Cross et al., 2001; Ougolkov et al., 2005; Rancillac et al., 2006; Gurrieri et al., 2010) side-by-side in both *in vivo* and *in vitro* assays in *Fmr1*^*KO*^ mice and cells, respectively. *In vivo* testing included AGS susceptibility, the gold standard test for drug efficacy evaluation in *Fmr1*^*KO*^ mice. Both MPEP and SB216763 significantly reduced wild running and seizures in *Fmr1*^*KO*^ mice, but the novel Angelini Pharma GSK3 inhibitor did not (Figure 2). The lower dose of AFC03127 was selected based on Angelini behavior data in *Fmr1*^*KO*^ mice where chronic treatment for 10–14 days with AFC03127 at 10 mg/kg significantly reduced marble burying (Angelini, personal communication), total distance traveled in the open field and startle response (Porceddu et al., 2021), but an acute 30 mg/kg treatment that significantly reduced total distance traveled in the open field appeared to cause sedation (Angelini, personal communication). AF03127 was tested at 30 mg/kg in *Fmr1*^*KO*^ mice in the AGS assay and exhibited a 29% seizure rate (*n* = 17) compared to 32% after treatment with 10 mg/kg (*n* = 22; data not shown, testing was with a separate set of control mice). Male and female mice were tested for all treatments; sex-specific effects were not observed.

**FIGURE 3:**
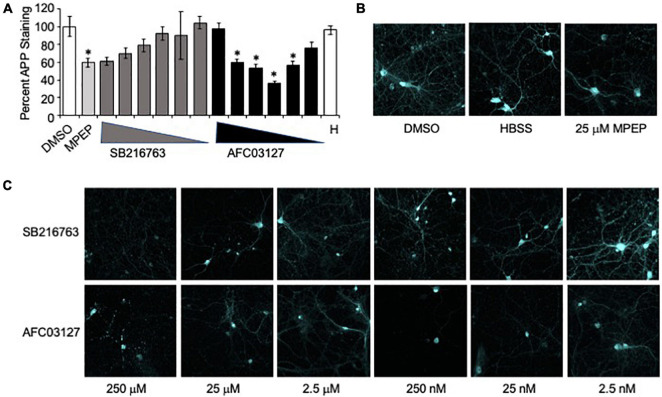
GSK3 inhibitors reduce neuronal APP expression in *Fmr1*^*KO*^ mice. **(A)** Primary cultured *Fmr1*^*KO*^ neurons were treated with MPEP (25 μM) versus SB216763 and AFC03127 over a concentration range of 2.5 nM–250 μM, stained with anti-APP antibody conjugated to Alexa Fluor^®^ 647 and images acquired with a 60x objective. Average APP staining intensities of 3–5 dendrites per cell for 9–18 cells (3–6 cells per slide, 3 slides per treatment) were plotted against drug treatment. Statistical significance was determined by ANOVA (*p* < 0.0001, *F* = 4.533, and dF = 14) with Dunnett’s *post hoc* multiple comparison test (Supplementary Table 1). Error bars represent SEM. Asterisks denote statistically significant differences in APP staining compared to vehicle-treated (*p* < 0.05). **(B)** Representative images are shown for vehicle, HBSS (H) and MPEP controls. **(C)** Representative images are shown of neurons treated with SB216763 and AFC03127.

The *in vitro* assays included dose response assessments of SB216763 and AFC03127 efficacy in reducing dendritic APP expression in *Fmr1*^*KO*^ primary neurons. There was a 40% reduction in dendritic APP staining in response to 25 μM MPEP (Figure 3). Testing a 100,000-fold concentration range, there was a statistically non-significant dose response decrease in dendritic APP staining in response to SB216763 with equivalent staining to MPEP at the highest dose of 250 μM SB216763 (Figure 3). A bell-shaped curve was observed in response to AFC03127 treatment such that APP staining was equivalent or lower with 25 nM–25 μM AFC03127 compared to MPEP (Figure 3 and Supplementary Table 1). The lowest effective *in vitro* dose was 25 nM AFC03127. The highest dose of AFC03127 (250 μM) did not reduce APP levels likely because the drug visibly precipitated out of solution forming a fine suspension, which may have settled on the cells in a localized high concentration causing cell death as evidenced by the shorter dendrites and “hot spots” of stained clumped puncta (Supplementary Figure 1). A greater than 60% reduction in dendritic APP was observed with 250 nM AFC03127 compared to prior studies demonstrating a maximal reduction of 50% APP with 250 nM AFQ-056, an mGluR_5_ inhibitor (Westmark et al., 2018).

**FIGURE 4:**
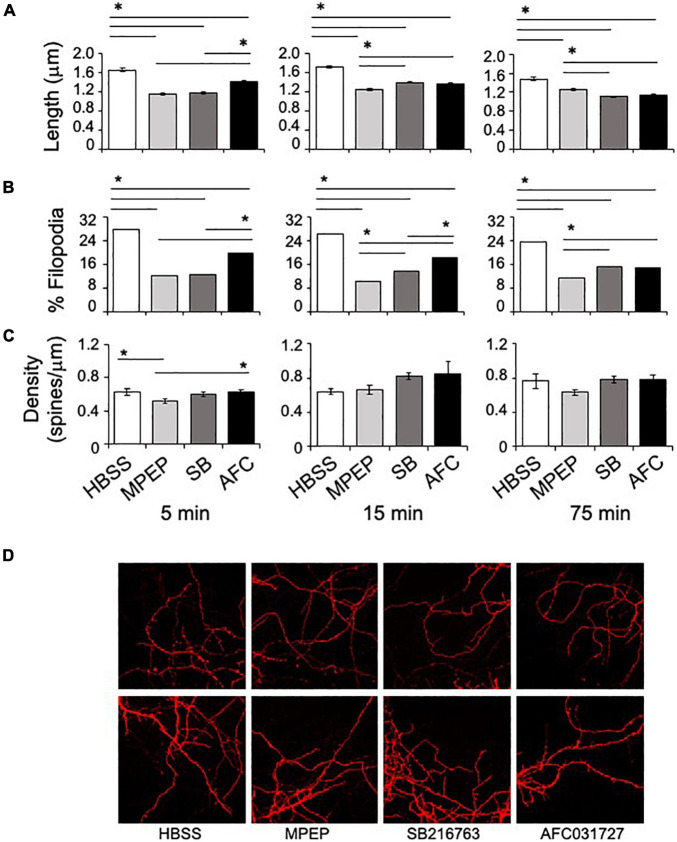
GSK3 inhibitors rescue dendritic spine morphology phenotypes in *Fmr1*^*KO*^ mice. **(A)** Primary cultured *Fmr1*^*KO*^ neurons were treated with 25 μM MPEP versus 25 μM SB216763 and 250 nM AFC03127 for 5, 15, or 75 min, stained with DiI and images acquired with an 100x objective. The lengths of dendritic protrusions were quantitated with ImageJ software and plotted against compound treatment. Statistical significance was determined by ANOVA [5 min (*p* < 0.0001, *F* = 101.8, and dF = 3); 15 min (*p* < 0.0001, *F* = 67.61, and dF = 3); and 75 min (*p* < 0.0001, *F* = 69.17, and dF = 3)]. Error bars represent SEM. Asterisks denote statistically significant differences by *post hoc* Tukey tests (*p* < 0.001). **(B)** The percentage of filopodia was plotted against compound treatment. Filopodia were defined as protrusions with a width-to-length ratio less than or equal to 0.5. Statistical significance was determined by Chi square analyses (4 × 2 tables) [5 min (*p* < 0.00001, Chi statistic = 191); 15 min (*p* < 0.00001, Chi statistic = 165); and 75 min (*p* < 0.00001, Chi statistic = 101)]. Asterisks denote statistically significant differences by Chi square analyses (2 × 2 tables; *p* < 0.003). **(C)** Spine density (# of spines per length of spine) was plotted as a function of compound treatment. Multiple areas of multiple cells were assessed for each treatment. Statistical significance was determined by ANOVA [5 min (*p* = 0.018, *F* = 3.38, and dF = 3); 15 min (*p* = 0.14, *F* = 1.85, and dF = 3); and 75 min (*p* = 0.18, *F* = 1.63, and dF = 3)]. Error bars represent SEM. Asterisks denote statistically significant differences by *post hoc* Tukey tests (*p* < 0.05). **(D)** Representative images from 5 min time point at 100x are shown.

The *in vitro* assays also included testing SB216763 and AFC03127 efficacy in rescuing dendritic spine length and density and percent filopodia in *Fmr1*^*KO*^ primary neurons at the optimal doses determined in the dendritic APP staining study. A single concentration of each drug was tested at 3 time points (5, 15, and 75 min). MPEP (25 μM), SB216763 (25 μM), and AFC03127 (250 nM) significantly reduced dendrite length compared to vehicle (Figure 4, Supplementary Figure 2, and Supplementary Table 2). The control *Fmr1*^*KO*^ cells exhibited an average spine length of 1.6 μm as expected, which is about two-fold longer than spines from wild type neurons (Westmark et al., 2011). With the 5-min treatment, MPEP and SB216763 reduced dendrite length to a greater extent than AFC03127. With the longer 15- and 75-min treatments, all drugs significantly reduced dendrite length compared to vehicle; however, the cells treated for longer times with GSK3 inhibitors were starting to die as evidenced by shrinking dendrites (data not shown). Similar to dendrite length, the percent filopodia were significantly reduced with MPEP, SB21673, and AFC03127 compared to vehicle (Figure 4). Spine density was significantly different by ANOVA for the 5-min treated samples (vehicle, MPEP, SB216763, and AFC03127), but *post hoc* tests did not show differences between either GSK3 inhibitor or the vehicle (Figure 4). Spine density was not significantly different by ANOVA for the 15- and 75-min treatments.

## Discussion

This study compared the efficacy of a novel Angelini Pharma GSK3 inhibitor AFC03127 with MPEP and SB216763 in the *Fmr1*^*KO*^ mouse model. The *Fmr1*^*KO*^ mice are the most widely employed animal model used for the study of FXS. They have good face validity in terms of exhibiting many of the physical and behavioral characteristics of human patients such as lower seizure threshold and abnormal dendritic spine morphology, even if they may be over predictive in terms of translating drugs to the clinic (Berry-Kravis et al., 2018). MPEP is a research grade mGluR_5_ antagonist that reduces AGS, anxiety and dendritic spine protrusion phenotypes in *Fmr1*^*KO*^ mice (Yan et al., 2005; de Vrij et al., 2008). SB216763 is a potent maleimide compound with ATP-competitive inhibitor activity against both GSK3α and GSK3β (Coghlan et al., 2000; Cross et al., 2001; Ougolkov et al., 2005; Gurrieri et al., 2010). AFC03127 was compared to MPEP and SB216763 in both *in vivo* and *in vitro* assays. The assays under evaluation included AGS, dendritic APP expression and dendritic spine measurements. These assays were chosen because they were previously shown to be altered in the *Fmr1*^*KO*^ mouse and responsive to treatment. Specifically, genetic and pharmaceutical modulation of APP levels rescued AGS, spine length and the percent filopodia (Westmark et al., 2011, 2018).

SB216763 (30 mg/kg) rescued the AGS phenotype in *Fmr1*^*KO*^ mice as well as MPEP, which corroborates prior findings (Min et al., 2009). AFC03127 did not rescue the AGS phenotype nor did tideglusib (200 mg/kg oral gavage; data not shown), which is an irreversible, non-ATP competitive GSK-3β inhibitor (Dominguez et al., 2012). AFC03127 significantly reduced dendritic APP expression over a wide concentration range whereas SB216763 only approached statistical significance at the highest dose. Both SB216763 and AFC03127 significantly rescued spine length and the percent filopodia in cultured *Fmr1*^*KO*^ neurons. SB216763 and AFC03217 equally inhibit both GSK3α and GSK3β (Coghlan et al., 2000; Furlotti et al., 2015, Angelini, personal communication). Inhibition of GSK3α but not GSK3β corrected excessive protein synthesis, mGluR-LTD, AGS, sensory cortex hyperexcitability, learning and memory, and enhanced psychomimetic-induced hyperlocomotion in *Fmr1*^*KO*^ mice (McCamphill et al., 2020). GSK3 mediates phosphorylation of the cytoplasmic domain of APP and processing of APP to amyloid-beta (Aβ; Aplin et al., 1996; Phiel et al., 2003; Su et al., 2004). Chronic GSK3 inhibition with lithium (non-selective), BRD0705 (α-selective), or BRD3731 (β-selective) reduced phosphorylation of APP but did not affect total APP levels (McCamphill et al., 2020). GSK3β activity increases the number of thin spines whereas lack of GSK3β increases the number of stubby spines (Kondratiuk et al., 2017). SB216763 and AFC03217 are not paralog selective inhibitors of GSK3α, albeit paralog-specific effects may underlie the varied AGS and dendritic findings.

The lowest effective dose of AFC03127 in our *in vitro* study was 1,000-fold lower than that for SB216763. We observed a bell-shaped dose response effect with AFC03127, in the reduction of dendritic APP levels, that could be due to a solubility issue or toxicity at higher concentrations of the drug. It is also feasible that there is a true bell-shaped dose response as both increased and decreased expression of *Drosophila* GSK3 impair larval crawling in the fly model of FXS (Jakubowski et al., 2012), suggesting that complex biological effects are associated with this enzyme. It is of interest that SB216763 but not AFC03127 attenuated AGS while AFC03127 but not SB216763 reduced dendritic APP expression and both drugs affected dendritic spine phenotypes. Recent work demonstrates that *Fmr1* deletion in glutamatergic neurons in the inferior colliculus is necessary to cause AGS (Gonzalez et al., 2019). The effects of GSK3 inhibitors in the inferior colliculus remain to be determined.

Drug development is expensive. and it is difficult to attract pharmaceutical companies to the study of rare disorders. Thus, when drugs can be repurposed for multiple disorders, it benefits both industry and the affected families. It is important to identify and test novel GSK3 inhibitors as a potential therapeutic strategy for FXS. Lithium has been widely used in the clinic and demonstrated early efficacy in preclinical models of FXS; however, this drug can have off-target effects and issues with tolerability in humans, while cessation of treatment leads to reemergence of phenotypes in *Fmr1*^*KO*^ mice (King and Jope, 2013). Herein, we provide preclinical validation data comparing the efficacy of AFC03127 with MPEP and SB216763 in *Fmr1*^*KO*^ mice. A limitation of the work is that there is not dose response data for the seizure study due to the large number of mice required and the decision to screen multiple compounds versus multiple doses. The data contribute to a rapidly growing body of preclinical data regarding the efficacy of GSK3 inhibitors in the rescue of *Fmr1*^*KO*^ phenotypes. In addition, GSK3 activation affects all the major hallmarks of Alzheimer’s disease (Cai et al., 2012; Lauretti et al., 2020). Thus, the development and validation of novel GSK3 inhibitors may benefit multiple CNS disorders. It will be of interest to study clinically relevant behavioral alterations in mouse models of FXS and Alzheimer’s disease in response to AFC03127 in future studies, particularly considering the pivotal role of GSK3 in APP biology and the wide range of phenotypes rescued in *Fmr1*^*KO*^ mice in response to GSK3 inhibitors (Rizk et al., 2021; Figure 1).

In conclusion, inhibitors of GSK3 are under investigation for the treatment of a wide range of CNS disorders. Regarding FXS, GSK3 inhibitors have shown promise in fly, mouse and human models. Herein, we compared the efficacy of a novel inhibitor of GSK3 developed by Angelini Pharma, AFC03127, with SB216763. SB216763 was effective in attenuating seizures *in vivo* in a mouse model useful for the study of FXS. AFC03127 were effective at attenuating APP biomarker expression and both drugs were effective in attenuating dendritic spine phenotypes in *in vitro* assays. AFC03127 was more effective than MPEP or SB216763 in reducing dendritic APP expression. These findings support further preclinical study of this family of GSK3 inhibitors for the treatment of FXS, Alzheimer’s disease and other APP/Aβ-related disorders.

## Data Availability Statement

The raw data supporting the conclusions of this article will be made available by the authors, without undue reservation.

## Ethics Statement

The animal study was reviewed and approved by University of Wisconsin Madison Institutional Animal Care and Use Committee (IACUC).

## Author Contributions

CW and PW conceived and designed the experiments, acquired data, and interpreted data. BG provided the test drugs. CW drafted the manuscript. CW, BG, PW, RO, CM, and FD edited and approved the manuscript. All authors agreed to be accountable for the work.

## Conflict of Interest

BG, RO, CM, and FD are employees of Angelini Pharma S.p.A. Angelini Pharma S.p.A. played no role in the design of the study or the collection and analysis of the data. The remaining authors declare that the research was conducted in the absence of any commercial or financial relationships that could be construed as a potential conflict of interest.

## Publisher’s Note

All claims expressed in this article are solely those of the authors and do not necessarily represent those of their affiliated organizations, or those of the publisher, the editors and the reviewers. Any product that may be evaluated in this article, or claim that may be made by its manufacturer, is not guaranteed or endorsed by the publisher.
