# Supplementary material for: Testing Fmr1KO Phenotypes in Response to GSK3 Inhibitors: SB216763 versus AFC03127
Source: Front Mol Neurosci. 2021 Oct 7;14:751307. doi: 10.3389/fnmol.2021.751307 (PMC8529056; doi:10.3389/fnmol.2021.751307)
Supplement: Supplementary file 1 [file Data_Sheet_1.PDF]

Supplementary Material

1 Supplementary Figures and Tables

Supplementary Figure 1: GSK3 inhibitor AFC03127 at 250 mM caused cell death. *Fmr1*^{KO} primary cultured neurons were treated with vehicle (left) versus AFC03127 (250 mM) (right), stained with anti-APP antibody conjugated to Alexa Fluor® 647 and images acquired with a 60x objective. The high dose of AFC03127 visibly precipitated out of solution forming a fine suspension, which may have caused the cells to start to die as evidenced by the shorter dendrites and "hot spots" of stained clumped puncta.

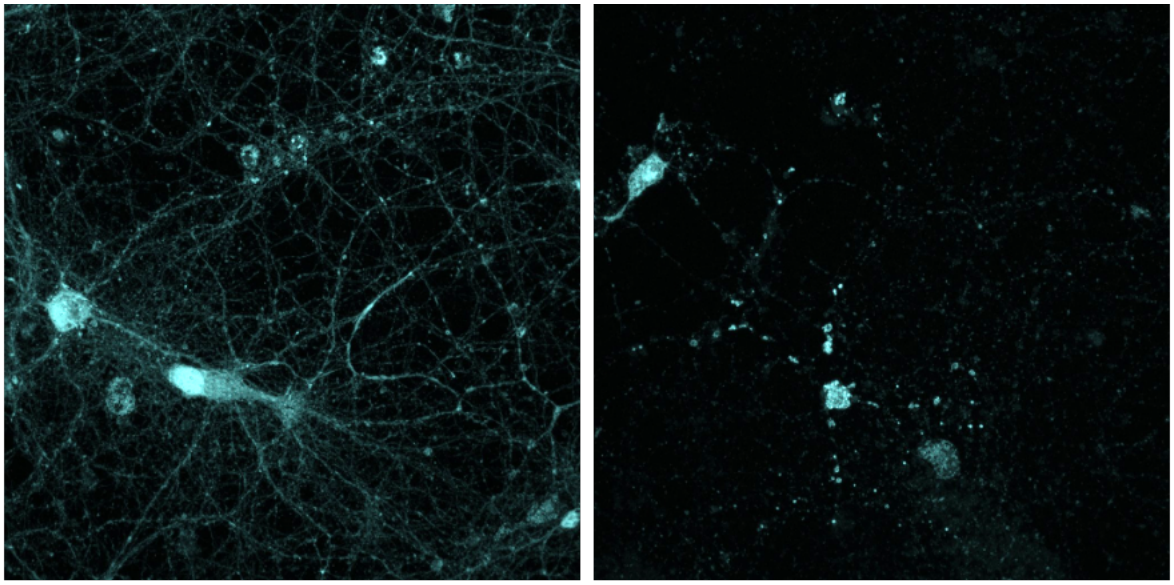

Vehicle   250 µM AFC03127

Supplementary Figure 2: *Fmr1*^{KO} primary cultured neurons can be stained with DiI to visualize filopodia and spines. **(A)** An example of a stained neuron at 20x and 100x. **(B)** An example of the contour drawings on a segment of dendrite.

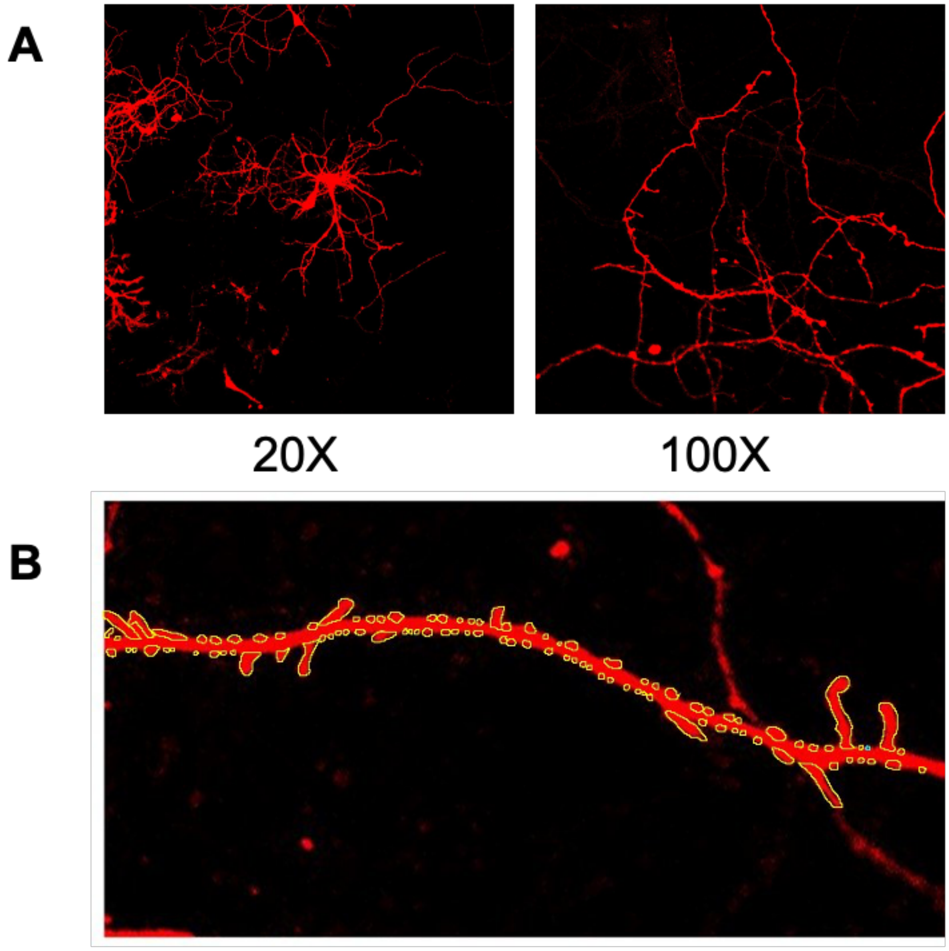

Supplementary Table 1<br>Confocal Data with Statistics<br>(*p* values compared to vehicle control)				
	**SB216763**		**AFC03127**	
**Dose**	**% APP Staining**	***p***	**% APP Staining**	***p***
250 μM	60.9	0.0696	97.2	0.9997
25 μM	69.9	0.2718	59.9	0.0410
2.5 μM	79.0	0.6577	53.9	0.0062
250 nM	91.9	0.9990	36.5	0.0002
25 nM	90.0	0.9954	56.8	0.0115
2.5 nM	104.4	0.9996	75.8	0.4194
Vehicle	100.0	--	100	--
25 μM MPEP: 59.5%, *p*=0.0342				
HBSS control: 96.1%, *p*=0.9996				

Supplementary Table 2: Dendritic Spine Analysis			
**Treatment**	**# Filopodia**	**# Spines**	**Total Projections**
HBSS 5 min	481	1252	1733
HBSS 15 min	415	1178	1593
HBSS 75 min	403	1296	1699
MPEP 5 min	207	1490	1697
MPEP 15 min	166	1477	1643
MPEP 75 min	190	1479	1669
SB216763 5 min	222	1564	1786
SB216763 15 min	271	1691	1962
SB216763 75 min	274	1515	1789
AFC03127 5 min	382	1558	1940
AFC03127 15 min	308	1398	1706
AFC03127 75 min	218	1238	1456
